# Purification, Characterization, and Potential Immune-Regulation Mechanism of Polysaccharides from *Artemisia odosica* Krasch.

**DOI:** 10.3390/molecules30030675

**Published:** 2025-02-03

**Authors:** Yuanyuan Xing, Yankai Zheng, Jing Zhang, Lu Chen, Yuanqing Xu, Xiao Jin, Lei Hong, Sumei Yan, Binlin Shi

**Affiliations:** 1 College of Animal Science, Inner Mongolia Agricultural University, Hohhot 010018, China; xingyuanyuan2014@163.com (Y.X.); 13848911743@163.com (Y.Z.); xuyuanqing@imau.edu.cn (Y.X.); yaojinxiao@aliyun.com (X.J.); hl1116@imau.edu.cn (L.H.); yansmimau@163.com (S.Y.); 2 Intellectual Property Protection Center of Inner Mongolia Autonomous Region, Hohhot 010000, China; jingyuer96@163.com; 3 Animal Husbandry and Veterinary Department, Shanxi Animal Husbandry and Veterinary School, Taiyuan 030024, China; 17835115282@163.com

**Keywords:** *Artemisia ordosica* Krasch. polysaccharides, purification, immune-regulation, NF-κB signaling pathway

## Abstract

*Artemisia ordosica* Krasch. represents a medicinal species traditionally and extensively employed in traditional medicine for treating ailments such as rheumatic arthritis, sore throat, and inflammation. This study initially focuses on the extraction, purification, and characterization of *Artemisia ordosica* Krasch. polysaccharides (AOP). The purified AOP exhibits a molecular mass corresponding to 9.00 kDa and consists of multiple monosaccharide units, with glucose (54.08%) as the predominant component, followed by arabinose (13.75%), mannose (13.43%), galactose (12.79%), xylose (3.15%), glucuronic acid (0.93%), galacturonic acid (0.67%), ribose (0.63%), and fucose (0.56%), respectively. Furthermore, to explore the immune-regulatory mechanisms of AOP, peripheral blood lymphocytes (PBLs) were cultured and exposed to inhibitors targeting receptors and signaling molecules. The results indicated that TLR4 serves as a potential target through which AOP exerts its immunomodulatory functions. AOP mitigates immune stress in PBLs triggered by LPS by disrupting the interaction between LPS and TLR and downregulating the over-activation of the nuclear factor kappa B (NF-κB) signaling pathway. In summary, AOP shows promise as a feed additive to protect animals from immune stress.

## 1. Introduction

Natural polysaccharides have emerged as crucial modulators of biological responses [1], exerting antinociceptive, anti-inflammatory, antioxidant and antitumor effects [2,3,4]. Recent studies have highlighted their significance as prebiotics, particularly due to their capacity to influence gut microbiome composition [5,6]. Hence, polysaccharides are widely used in the materials, pharmaceutical, and nutritional sectors [7], though their implementation in livestock production systems still requires a great deal of data support. The plant *Artemisia ordosica* Krasch. is abundant in nutrients and encompasses diverse bioactive constituents including polysaccharides, flavonoids, essential oils, and triterpenoid compounds. In our previous studies, polysaccharides, being the principal bioactive constituents of *Artemisia ordosica* Krasch., demonstrated multiple physiological benefits, including enhanced growth performance in broilers, improved intestinal structure, and elevated digestive enzyme activities [8,9,10]. Nevertheless, further investigation into their activities through isolation and purification processes is necessitated. The bioactivity of these complex carbohydrates correlates strongly with their structural features, including size distribution, sugar unit composition, and linkage patterns [11]. Given the inherent complexity of plant polysaccharides, multiple purification steps are essential to remove impurities like pigments, proteins, and phenolic substances. Accurate characterization and quantification of polysaccharides and their components are crucial in *Artemisia ordosica* Krasch. polysaccharides (AOP) research. In this study, ion-exchange chromatography and gel permeation chromatography were employed for additional separation and purification of AOP. Subsequently, components demonstrating potent immunomodulatory were isolated to elucidate them underwent subsequent in vitro lymphocyte culture investigations.

Polysaccharides primarily regulate immune cell function and metabolism by interacting with polysaccharides receptors on the surface of immune cells, initiating downstream cascade reactions through various intracellular signaling pathways [12,13,14]. Key receptors involved in polysaccharides recognition include pattern recognition receptors such as TLRs, CR3, the Dectin-1 molecule, mannose-binding proteins, scavenger recognition molecules, and membrane immunoglobulin complex receptor [15]. While TLR4 is known to bind diverse extracellular signals that activate immune cells [16], its specific interaction with AOP requires further investigation. Therefore, this study aims to explore whether TLR4 serves as a target receptor for AOP and its implications in the presence of LPS-whether synergistically or otherwise. Active polysaccharides have been reported to protect immune cells from structural damage caused by external stimuli and enhance the intracellular antioxidant enzyme system via TLR4-mediated nuclear factor kappa B (TLR4/NF-κB) signaling pathway, which modulates inflammatory mediator expression in immune cells [17,18]. The NF-κB pathway plays a central role in orchestrating inflammatory responses, acting as a transcriptional regulator composed of Rel protein homodimers or heterodimers [19]. Previous research has demonstrated the significant immunomodulatory and antioxidant properties of AOP in a broiler chicken immune stress model [9,10]. Inhibiting NF-κB activation to suppress pro-inflammatory cytokine production is a potential mechanism through which many active polysaccharides exert their immunomodulatory effects. However, a comprehensive understanding of the precise underlying mechanisms is essential. In this study, we screened 150 μg/mL AOP as the optimal concentration to investigate its immune-antioxidant modulation mechanism in vitro. We employed in vitro lymphocyte cell culture assays and a TLR4/NF-κB pathway inhibitor blockade assay using the highly specific NF-κB P65 inhibitor PDTC to verify whether AOP mitigates immune stress in broiler lymphocytes via the TLR4/NF-κB signaling pathway.

## 2. Results and Discussion

### 2.1. Purification of AOP

**Figure 1:**
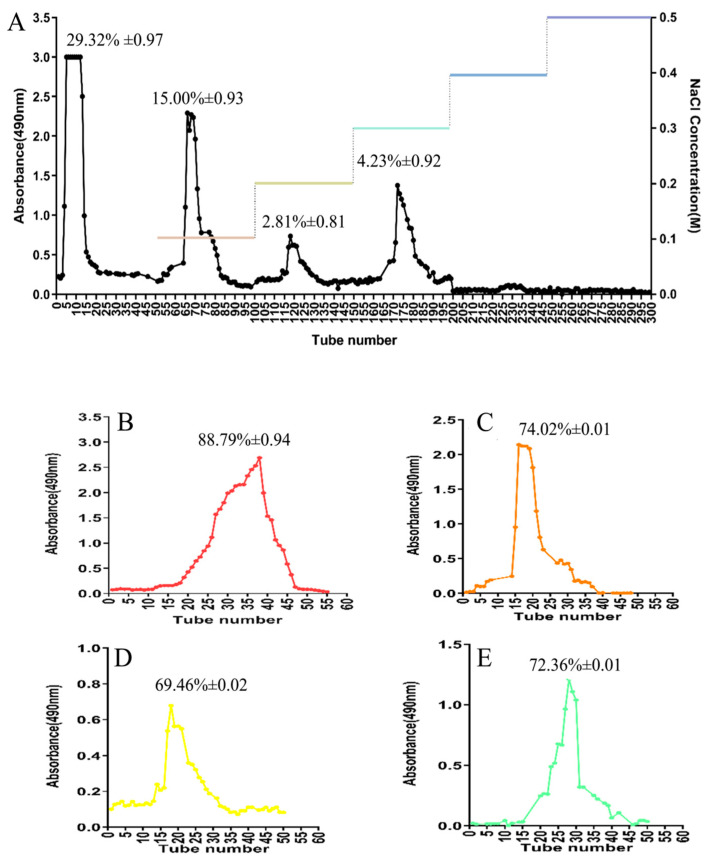
Fractionation of the AOP extract by DEAE-52 and Sephadex G-100 chromatography. Note: (**A**): elution profile of the crude AOP extract on a DEAE-52 anion exchange column, the yields of polysaccharides elution with distilled water, 0.1 M NaCl, 0.2 M NaCl, and 0.3 M NaCl were 29.32%, 15.00%, 2.81%, and 4.23% of the total AOP, respectively. (**B**–**E**): Elution profiles of fractions from the DEAE column after further fractionation on a Sephadex G-100 size-exclusion column, (**B**–**E**) corresponded to elution with distilled water (red color), 0.1 M NaCl (orange color), 0.2 M NaCl (yellow color), and 0.3 M NaCl (green color) with total sugar contents were 88.79%, 74.02%, 69.46%, and 72.36%, respectively.

In recent years, the purification of polysaccharides has primarily involved the use of anion-exchange cellulose column chromatography [20,21]. The polysaccharides from *Artemisia ordosica* Krasch. underwent purification through anion exchange chromatography using a DEAE-52 column chromatography. This process resulted in the separation of four distinct elution peaks. These peaks corresponded to elution using sequential solutions: deionized water followed by increasing sodium chloride concentrations (0.1, 0.2, 0.3, 0.4, and 0.5 M), as illustrated in Figure 1A. Each elution peak was collected, concentrated, dialyzed, and subjected to vacuum freeze-drying to obtain pure polysaccharides. The yields of polysaccharides obtained from the sequential elution’s deionized water and increasing NaCl concentrations were 29.32%, 15.00%, 2.81%, and 4.23% of the total AOP, respectively. While DEAE-cellulose column chromatography has been widely used for the isolation and purification of plant polysaccharides, it is worth noting that this method has a relatively slow elution rate, longer elution times, and a reduction in column bed height as the ionic strength of the eluting solution increases [21]. Recent advancements have seen researchers continuously developing novel anion-exchange agents and gel resins with desirable features [22]. These advancements present promising avenues for future separation and purification of AOP.

Following ion-exchange column chromatography, active polysaccharides often exhibit non-uniform molecular weights. To achieve polysaccharides with uniform molecular weights, gel permeation chromatography (GPC) is employed. Hence, each component obtained from the initial anion exchange separation underwent subsequent purification through size exclusion using a Sephadex G-100 chromatography, based on molecular sieving effects, as illustrated in Figure 1B–E. After separation and purification, single elution peaks were obtained. This observation indicates successful purification, yielding polysaccharides of uniform molecular weight for all individual components. These peaks were collected, concentrated, subjected to dialysis and lyophilization to yield purified AOP fractions. The total sugar contents of the fractions eluted sequentially with deionized water and increasing NaCl gradient were 88.79%, 74.02%, 69.46%, and 72.36%, respectively. Due to the high yield and sugar purity of polysaccharides eluted with distilled water, we prioritized the detailed structural characterization of this major neutral polysaccharide fraction, designated as AOP. The structural features of AOP were extensively characterized through multiple analytical approaches, including molecular mass determination, monosaccharide profiling, and spectroscopic analysis. Previous studies have established that aqueous solubility significantly influences polysaccharide bioactivity [11,23]. At 25 °C, AOP exhibits solubility in deionized water, laying the foundation for its biological activity expression. In summary, these results collectively indicate that DEAE-cellulose column chromatography coupled with Sephadex G-100 technology is effective for the isolation and purification of AOP, resulting in bioactive AOP.

### 2.2. Molecular Weight, Monosaccharide Composition, and UV-Spectra of AOP

**Figure 2:**
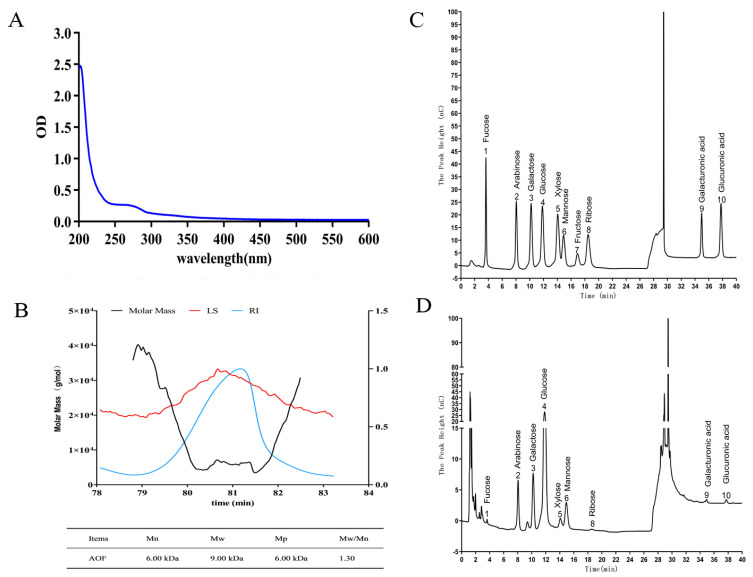
Molecular weight, monosaccharide composition and UV-spectra of AOP. Note: (**A**): UV-spectra of AOP; (**B**): molecular mass determination of AOP obtained from GPC-MALS-RI; (**C**,**D**): monosaccharide composition of monosaccharide standards and AOP obtained from ion chromatography.

Spectrophotometric analysis of AOP was conducted across wavelengths spanning 200–600 nm, with results depicted in Figure 2A. It revealed a strong absorption peak at 200 nm, indicating a high polysaccharides content in AOP. The absence of characteristic absorbance peaks in the 260–280 nm region confirmed the successful removal of protein and nucleic acid contaminants during purification.

The RI-MALS chromatogram of AOP is shown in Figure 2B. From the chromatogram, a peak can be observed in the elution time range of 78.8–82.5 min. The average molecular weight (Mw) of AOP was determined to be 9.00 kDa, with a number-average molecular weight (Mn) of 6.00 kDa. The calculated polydispersity index (Mw/Mn) of 1.30 approached unity, suggesting a high degree of molecular uniformity in the purified fraction.

Analysis of monosaccharide constituents was performed through ion chromatographic separation, with results presented in Figure 2C,D. Based on the elution times of monosaccharide standards, it was determined that AOP was composed of the following monosaccharides: nine distinct sugar units including neutral and acidic monosaccharides. The composition analysis revealed glucose as the predominant component (54.08%), followed by arabinose (13.75%), mannose (13.43%), and galactose (12.79%), with minor proportions of xylose (3.15%), fucose (0.56%), ribose (0.63%), galacturonic acid (0.67%), and glucuronic acid (0.93%), indicating that AOP was a neutral polysaccharide. Research indicates that polysaccharides containing arabinose, mannose, and galactose often exhibit strong antioxidant activities, which is attributed to their impact on the conformation and higher structure of polysaccharides [24,25]. While these compositional findings establish a fundamental understanding, provides an initial characterization of the AOP, further extensive research is required to fully unravel its structural intricacies.

### 2.3. Immunomodulatory Effects of TLR4 as a Candidate Target of AOP

**Figure 3:**
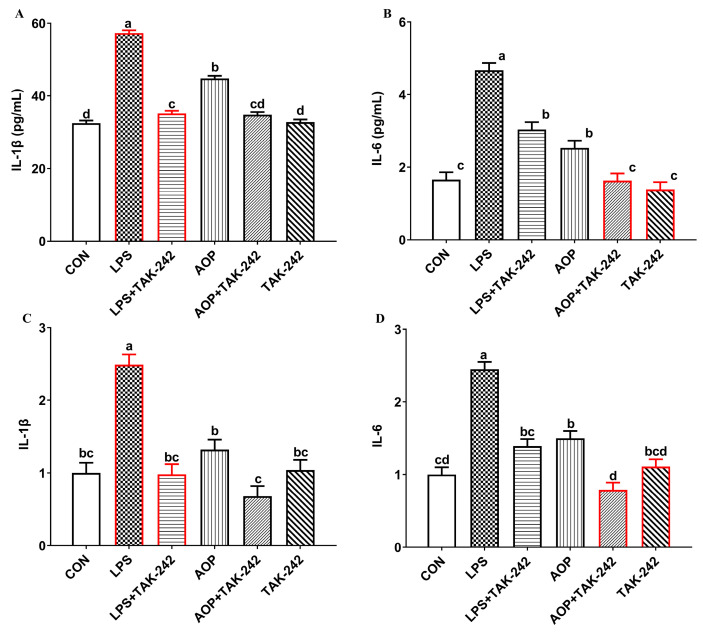
Effects of AOP on contents, gene expression of inflammatory cytokines in PBLs blocked by TAK-242. Note: (**A**,**B**): quantification of IL-1β and IL-6 protein levels in culture supernatants using enzyme-linked immunosorbent assays. (**C**,**D**): the relative mRNA expression of IL-1β and IL-6 determined by quantitative real-time PCR. Red lines represent groups treated with lipopolysaccharide. CON (Control group), LPS (lipopolysaccharides), AOP (*Artemisia ordosica* Krasch. polysaccharides), and IL (interleukin). each value is shown as mean ± SEM (n = 6). Different letters above bars indicate statistically significant differences between groups (*p* < 0.05).

Numerous studies have highlighted the initial step in polysaccharides-mediated cellular activity regulation as their binding to cellular receptors. In recent research, TLR4 has emerged as a crucial membrane receptor for polysaccharides [16,26]. Based on these findings and our experimental results, we hypothesized that AOP activates lymphatic cells through a specific cellular receptor. This prompted our investigation into whether TLR4 serves as a receptor for AOP. As shown in Figure 3, exposure to LPS induced a significant upregulation of both protein levels and transcript expression of IL-1β and IL-6 in PBL supernatants compared to untreated controls (*p* < 0.01). Pre-treatment with TAK242, a selective TLR4 antagonist, markedly attenuated these LPS-induced inflammatory responses (*p* < 0.01), confirming LPS signaling through TLR4. Similarly, AOP treatment elevated IL-1β and IL-6 expression at both protein and mRNA levels, though to a lesser extent than LPS (*p* < 0.01). The AOP-induced inflammatory response was also substantially reduced by TAK242 pre-treatment (*p* < 0.01). This indicates that TLR4 receptor blockade inhibits both LPS and AOP binding to lymphocytes, thereby reducing the rise in pro-inflammatory factors. Hence, TLR4 not only acts as a specific recognition receptor for LPS but also emerges as a candidate target for the immunomodulatory effects of AOP.

### 2.4. AOP Interfering with LPS Binding to TLR4

**Figure 4:**
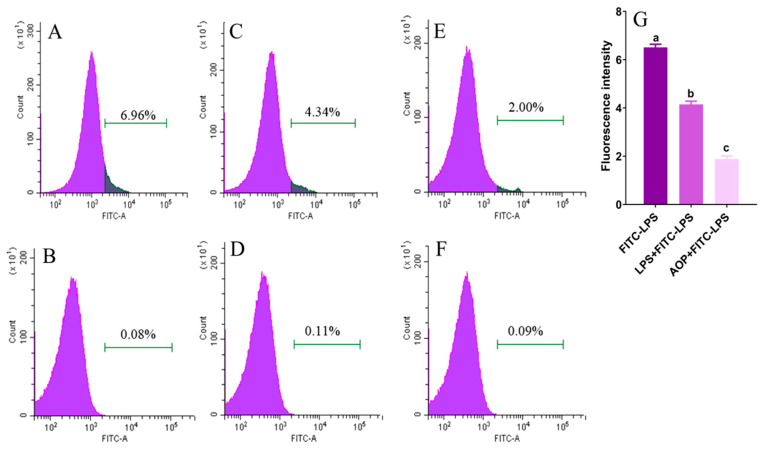
Competitive inhibition effects of AOP on receptors of PBLs combined with FITC-LPS. Note: (**A**): FITC-LPS group, (**B**): CON group, (**C**): LPS + FITC-LPS group, (**D**): LPS group, (**E**): AOP + LPS group, (**F**): AOP group, (**G**): average fluorescence intensity of LPS-FITC, LPS + LPS-FITC, and AOP + LPS-FITC groups. Each value is shown as mean ± SEM (n = 6). Different letters above bars indicate statistically significant differences between groups (*p* < 0.05).

LPS initiates intracellular signaling by binding to TLR4 on PBLs’ surfaces, triggering inflammatory mediator synthesis and release. Similarly, AOP exerts its mitigating effects by binding to TLR4 and subsequently regulating downstream target genes. This raises the question of their interaction when coexisting. To address this, we conducted a systematic study to elucidate the mechanism of AOP. We employed flow cytometry to examine AOP’s competitive inhibition of FITC-LPS binding to cell membrane surface receptors. Flow cytometric analysis demonstrating competitive receptor binding between AOP and fluorescein-labeled LPS on PBLs is presented in Figure 4. The highest fluorescence intensity of FITC-labeled LPS (LPS-FITC) was observed (*p* < 0.01), indicating strong binding. In the LPS + LPS-FITC group, the fluorescence intensity values of LPS-FITC-bound cells showed marked reduction relative to LPS-FITC alone, demonstrating that LPS competitively inhibited the binding of LPS-FITC to cellular receptors, resulting in decreased LPS-FITC binding to cells (*p* < 0.01). When AOP was co-administered with LPS-FITC (AOP + LPS-FITC), the difference in mean fluorescence intensity was also significantly reduced relative to the LPS-FITC control (*p* < 0.01), indicating that AOP interferes with LPS binding to cell-surface receptors, highlighting its potential to alleviate immune stress both in vivo and in vitro. Moreover, this reveals AOP’s bidirectional immunomodulatory effect: under normal conditions, AOP binds to TLR4, activating downstream pathways and slightly increasing pro-inflammatory factor production. However, during exogenous stress, AOP competes with harmful agents for TLR4 binding, thereby attenuating excessive pro-inflammatory factor production.

### 2.5. AOP Attenuates the Immune Stress Through Alleviates the Decrease in Cell Viability of PBLs Induced by LPS

**Figure 5:**
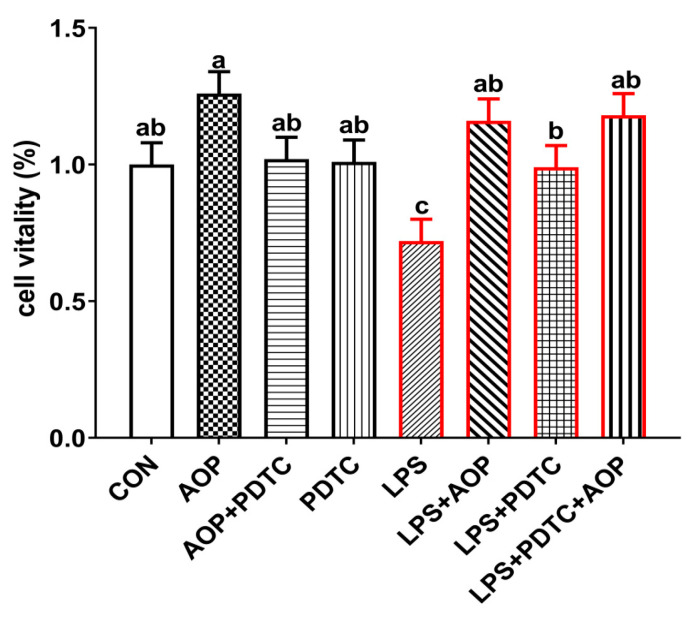
Effects of AOP on cell viability of PBLs challenged by LPS and blocked by PDTC (%). Note: CON (control group), LPS (lipopolysaccharide), AOP (*Artemisia ordosica* Krasch. Polysaccharides), and PDTC (NF-κB inhibitor). Red lines represent groups treated with LPS. each value is shown as mean ± SEM (n = 6). Different letters above bars indicate statistically significant differences between groups (*p* < 0.05).

Previous studies have demonstrated that polysaccharide-based compounds enhance immune cell proliferation and differentiation while maintaining low cytotoxicity [27,28,29]. In alignment with these findings, AOP significantly protected LPS-induced cytotoxicity in broiler chicken lymphocytes. As shown in Figure 5, the viability of PBLs decreased markedly following LPS treatment relative to untreated controls, whereas cell viability of LPS + AOP treated cells and the LPS + PDTC + AOP treated populations were significantly higher than LPS treatment alone (*p* < 0.05), while all other treatment groups had no significant effect on cell viability. Moreover, research suggests that polysaccharides compounds not only exert their mitigating effects by promoting the proliferation of immune cells but also by restoring changes in cell morphology induced by LPS. Xu et al. (2020) [30] found that when monocytes/macrophages were stimulated with LPS, these cells underwent morphological changes. The addition of *Astragalus* polysaccharides nanoparticles could directly act on immune cells, restore cell morphology, make cells return to their oval shape, improve cell membrane damage, and reduce extracellular fluid leakage, thus exerting anti-inflammatory effects. However, this study did not investigate the impact of AOP on changes in lymphocyte morphology under stress or non-stress conditions. Therefore, further studies are warranted to investigate how AOP can restore structural alterations in cell morphology under conditions of immune stress.

### 2.6. AOP Attenuates the Immune Stress in PBLs Induced by LPS Through Suppressing the Over-Expression of NF-κB Signaling Pathway

**Figure 6:**
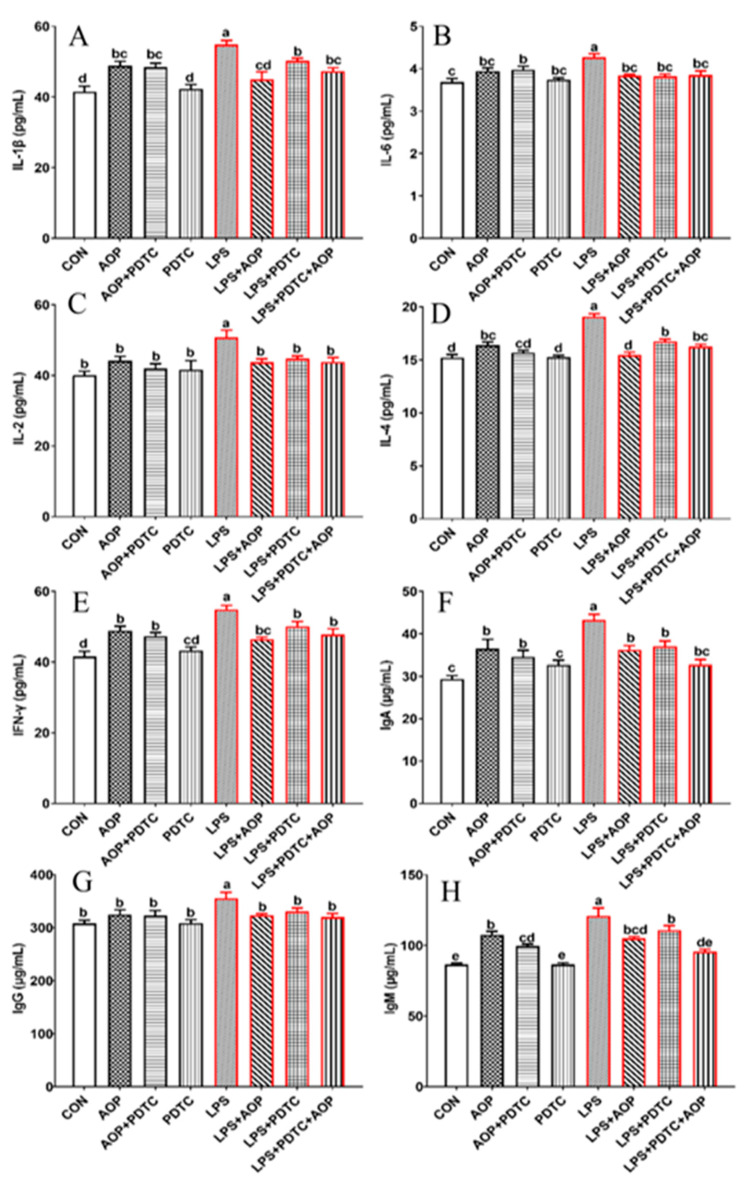
Effects of AOP on inflammatory cytokine and immunoglobulin contents in PBLs challenged by LPS and blocked by PDTC. Note: (**A**–**H**): quantification of IL-1β, IL-6, IL-2, IL-4, IFN-γ, IgA, IgG, and IgM protein levels in culture supernatants using enzyme-linked immunosorbent assays. Red lines represent groups treated with lipopolysaccharide. CON (Control group), LPS (lipopolysaccharide), AOP (*Artemisia ordosica* Krasch. polysaccharides), and PDTC (NF-κB inhibitor). Each value is shown as mean ± SEM (n = 6). Different letters above bars indicate statistically significant differences between groups (*p* < 0.05).

Figure 6 demonstrates that treatment with AOP alone led to significant increases in IL-1β, IL-6, IFN-γ, IgA, and IgM levels relative to untreated controls (*p* < 0.01). However, AOP + PDTC treatment resulted in lower values for these immune markers relative to the AOP-only treatment, suggesting that AOP modulates immune function through mild stimulation of pro-inflammatory mediator release under non-stress conditions, with PDTC partially attenuating these AOP-mediated effects. The PDTC-only treatment group showed no substantial alterations in any measured parameters.

Administration of LPS resulted in markedly elevated inflammatory mediators and immunoglobulin concentrations when compared to both control and AOP-treated samples. This LPS-induced elevation was attenuated in groups receiving concurrent treatment with AOP, PDTC, or their combination (LPS + AOP, LPS + PDTC, LPS + PDTC + AOP). Notable differences were observed in cells treated with LPS + AOP + PDTC, which showed significantly reduced IL-1β production relative to PDTC + LPS treatment, while displaying enhanced IFN-γ production relative to the LPS + AOP treatment condition (*p* < 0.05).

Our experimental results revealed that LPS administration led to increased secretion of pro-inflammatory cytokines (IL-1β, IL-6, IL-2, IL-4, IFN-γ) and immunoglobulins (IgA, IgG, and IgM) in the culture medium of PBLs, which were consistent with previous studies [31]. AOP treatment alone enhanced the production of specific immune mediators, with PBLs showing higher concentrations of IL-1β, IL-6, IFN-γ, IgA, and IgM compared with untreated controls. The current findings further reaffirm that AOP alleviate immune stress in vivo. As mentioned in the introduction, polysaccharides play a crucial role in immunomodulation by modulating the NF-κB pathway. Previous research has established that LPS induces NF-κB over-expression in macrophages, whereas blackberry polysaccharides significantly reduce its expression, thereby mitigating pro-inflammatory cytokine secretion [32]. While our earlier research demonstrated AOP’s ability to modulate immune stress in broilers through NF-κB pathway regulation, the detailed molecular mechanisms underlying this effect warrant further investigation.

**Figure 7:**
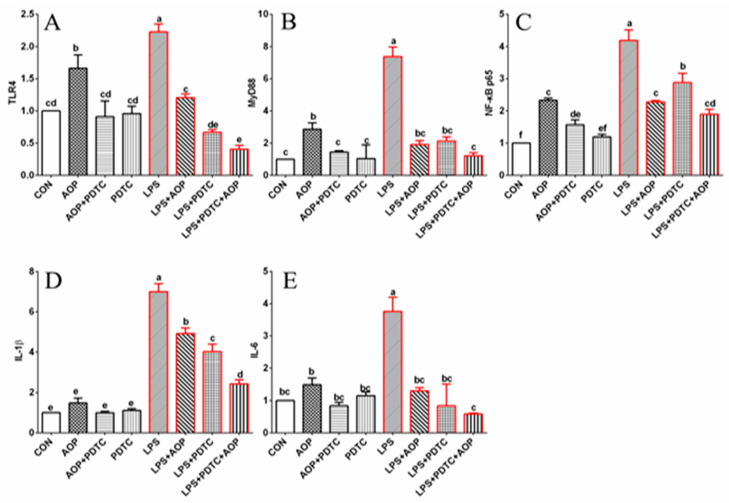
Effects of AOP on gene expression of NF-κB signal pathway in PBLs challenged by LPS and blocked by PDTC. Note: (**A**–**E**): the gene expression levels of TLR4 (toll like receptor 4), MyD88 (myeloid differentiation primary response 88), NF-κB p65 (nuclear factor kappa-B p65), IL-1β (interleukin-1β), and IL-6 (interleukin-6) were detected by qRT-PCR. Red lines represent groups treated with lipopolysaccharide. CON (control group), LPS (lipopolysaccharide), AOP (*Artemisia ordosica* Krasch. polysaccharides), and PDTC (NF-κB inhibitor). β-actin was used as a housekeeping gene. The relative expression levels from the control group were used as reference values. Each value is shown as mean ± SEM (n = 6). Different letters above bars indicate statistically significant differences between groups (*p* < 0.05).

To elucidate the role of TLR4/NF-κB signaling pathway in AOP-mediated immune modulation of broiler lymphocytes, we employed PDTC, a selective inhibitor of NF-κB P65 [33]. Our findings revealed that AOP treatment significantly increased the expression of TLR4, MyD88, and NF-κB P65 genes (*p* < 0.05, Figure 7). This AOP-induced upregulation was significantly diminished when cells were co-treated with AOP and PDTC (*p* < 0.05), while PDTC treatment alone showed no substantial effect on gene expression. Hence, AOP enhanced the expression of multiple components of the TLR4/NF-κB pathway, including TLR4, IKKβ, IκBα, NF-κB P65, and downstream cytokines IL-1β and IL-6. These AOP-mediated effects were effectively counteracted by PDTC co-treatment, supporting the involvement of TLR4/NF-κB pathway activation in AOP’s immunomodulatory function under non-stress conditions. The absence of significant changes in measured parameters following PDTC administration alone confirmed its neutral effect on cellular function. These results, together with existing evidence [34,35,36], suggest that botanical polysaccharides primarily function as immune system enhancers under non-stress conditions.

Gene expression analysis revealed that LPS treatment markedly upregulated NF-κB pathway components compared to both control and AOP-treated groups (*p* < 0.05). This LPS-induced upregulation was significantly attenuated by concurrent administration of AOP, PDTC, or their combination. Among them, the gene expression of NF-κB signaling pathway-related factors in the LPS + PDTC + AOP group was lower than that in the LPS + AOP and LPS + PDTC groups (*p* < 0.05). PDTC’s inhibitory effect on NF-κB P65 effectively reduced LPS-induced pro-inflammatory factor production [33]. Similarly, AOP co-treatment with LPS decreased both inflammatory mediators and expression of TLR4/NF-κB pathway components (TLR4, IKKβ, NF-κB P65, IL-1β, and IL-6). The NF-κB signaling cascade involves cytoplasmic sequestration of the P50/P65/IκBα complex until IKKβ-mediated phosphorylation triggers IκBα degradation, enabling nuclear translocation of NF-κB and subsequent immunoregulatory gene expression [37,38]. These findings suggest that AOP, like PDTC, functions by preventing excessive TLR4/NF-κB pathway activation during immune stress. Notably, the combined treatment (LPS + PDTC + AOP) demonstrated enhanced protective effects compared to either agent alone, indicating synergistic action between AOP and PDTC in counteracting LPS-induced immune stress.

In summary, a comprehensive analysis of prior research, combined with our experimental results, underscores the bidirectional regulatory effects of polysaccharides in the field of immunology [18,39,40]. Emerging evidence indicates that polysaccharides engage multiple signaling cascades to mediate their antioxidant effect and immune-regulation [15,41], and these signal pathways have complex crosstalk with TLR4/NF-κB signaling, potentially influencing NF-κB p65 nuclear translocation through multiple mechanisms. This intricate network of pathway interactions of polysaccharides by regulating NF-κB-mediated immune-regulation response. However, the precise mechanisms underlying these effects warrant further investigation.

## 3. Materials and Methods

### 3.1. Preparation of AOP

*Artemisia ordosica* Krasch. specimens were obtained from the Erdos region during July. The extraction of AOP followed protocols established in previous work [9]. The polysaccharides were isolated using aqueous extraction followed by ethanol-mediated precipitation. The plants, excluding roots, were cleaned with distilled water and naturally dried under shade at ambient temperature. The resulting plant material was ground to achieve passage through a 60-mesh sieve. The powder underwent a defatting process using 12 h Soxhlet extraction with petroleum ether. Subsequently, a 200 g portion of defatted material was immersed within distilled water under specific temperature and time conditions. The resulting solution underwent 0.45 μm filtration and was concentrated via rotary evaporation to 20% of the original volume. The concentrated extract was combined with anhydrous ethanol in a volume ratio of 4:1 (ethanol–extract), followed by polysaccharide precipitation at 4 °C over 48 h. The precipitated material underwent centrifugation at 12,000× *g* for 15 min, followed by successive washing steps using petroleum ether, acetone, and ethanol. The resulting precipitate underwent aqueous dissolution and protein removal using two treatments of Sevag reagent (n-butanol: chloroform = 1:4). The aqueous phase was subjected to dialysis against distilled water at 4 °C using a 500 Da molecular weight cutoff membrane (Beijing Solarbio Science and Technology Co., Beijing, China) for 48 h, replacing the dialysis medium every 12 h. The dialyzed solution underwent lyophilization to yield a white powder that was stored at −20 °C. For chromatographic purification involved packing DEAE-52 resin into a glass column, sterilizing with deionized water, and equilibrating with deionized water to achieve a 100 mL bed volume. A solution of 200 mg AOP in 20 mL distilled water was prepared. The column separation proceeded using sequential elution with distilled water followed by increasing NaCl concentrations (0.1, 0.2, 0.3, 0.4, and 0.5 M), maintaining a 1 mL/min flow rate. The total gradient elution time was 15 h, with 2.5 h for each step gradient. Fractions of 150 mL were collected from each elution step. The sugar content of AOP was quantified by phenol–sulfuric acid assay, and elution profiles were plotted based on absorbance values. Six components eluted with water and 0.1–0.5 M NaCl solutions were concentrated, desalted by dialysis, and freeze-dried. Weigh 25 mg of each sample component and dissolve them in 5 mL of ultrapure water. Following complete dissolution, the samples were loaded onto a chromatography column filled with Sephadex G-100 gel. Ultrapure water served as the mobile phase for washing, at 0.5 mL/min. Automated collection was facilitated to gather 5 mL fractions. Polysaccharides content within each fraction were determined using the phenol–sulfuric acid method and plotted as constructed. Following chromatographic profile, purified polysaccharides solutions were isolated, concentrated, subjected to desalination via dialysis, and ultimately freeze-dried. The purified polysaccharides components were stored at −20 °C until further analysis.

### 3.2. Characterization of AOP

#### 3.2.1. Molecular Weight

For molecular weight analysis, AOP was dissolved in water (5 mg/mL), heated to 100 °C for 5 min, and centrifuged (12,000× *g*, 10 min) to obtain a clear supernatant. Molecular weight characterization was performed using a GPC–MALLS–RI system. The analysis employed a mobile phase containing 0.1 M NaNO_3_ and 0.02% (*w*/*v*) NaN3, operated at 0.4 mL/min flow rate and 60 °C column temperature. The sample injection volume was set at 20 μL. The system provided measurements of weight-average molecular weight (Mw), number-average molecular weight (Mn), polydispersity index (Mw/Mn), and root-mean-square radius (R) for the AOP sample.

#### 3.2.2. Monosaccharide Composition

The monosaccharide composition analysis began with hydrolyzing 5 mg AOP in 4 mL of 2 M trifluoroacetic acid (TFA) within a sealed ampoule at 121 °C for 2 h. Following hydrolysis, TFA was removed via rotary evaporation. The resulting residue underwent three cycles of dissolution in methanol (2 mL) and evaporation under nitrogen stream. The final residue was dissolved in ultrapure water and transferred to a chromatography vial. Monosaccharide analysis was performed using ion-exchange chromatography (Dionex ICS 5000, Sunnyvale, CA, USA) with a CarboPac PA20 column (250 × 4 mm; Dionex). The elution profile consisted of three phases: 1% 500 mM NaOH (0–25 min), followed by 10% 500 mM NaOH with 90% ultrapure water (25.1–40 min), and finally 100% 500 mM NaOH (40.1–50 min). The analysis was conducted at 30 °C with a 0.5 mL/min flow rate and 20 μL injection volume. Standard monosaccharides included rhamnose, arabinose, galactose, glucose, xylose, mannose, fructose, ribose, galacturonic acid, and glucuronic acid.

#### 3.2.3. UV Spectral Scanning Analysis

Prepare an adequate quantity of water-washed AOP sample, ensuring it is completely solubilized in 1 mL of sterilized water. After preparation, subject this prepared sample to analysis by initiating scanning with an initial wavelength set at 200 nm, concluding at 600 nm, and maintaining a scanning interval of 1 nm.

### 3.3. Isolation and Culture of Peripheral Blood Lymphocytes

Peripheral blood was drawn from wing veins of male broiler chickens (42-day-old) using heparinized collection tubes. The isolation and culture protocol for peripheral blood lymphocytes (PBLs) was adapted from Tariq (2015) [42]. Blood samples (5 mL) were carefully layered over an equal volume of lymphocyte separation medium. Following centrifugation (2500 rpm for 20 min), the distinct lymphocyte layer was harvested and collected 15 mL tubes. The isolated cells underwent two PBS washing cycles before resuspension in RPMI-1640 medium supplemented with 10% fetal bovine serum and 1% penicillin-streptomycin. Cell viability and concentration were assessed using Trypan Blue staining (TBD, Tianjin), and the suspension was diluted to 2 × 10^6^ cells/mL. The prepared lymphocytes were distributed into 24-well sterile culture plates at 2 mL per well.

### 3.4. Treatment of PBLs with Receptor and Signaling Molecules Inhibitors

To study the immune-regulation mechanism of AOP, we first examined whether TLR4 serves as a target receptor for AOP. We designed 6 groups: (1) control group (CON), (2) LPS treatment group (LPS, administered at 10 μg/mL for 6 h), (3) LPS combined with TAK-242 treatment group (LPS + TAK-242, cells were pretreated with TAK-242 at a 100 nM dose for 0.5 h and then exposed to LPS at a 10 μg/mL dose for 6 h), (4) AOP treatment group consisting of 150 μg/mL AOP exposure for 24 h (AOP), (5) AOP combined with TAK-242 (AOP + TAK-242, cells were pretreated with TAK-242 at a 100 nM dose for 0.5 h and then treated with AOP at a 150 μg/mL dose for 24 h), and (6) TAK-242 group (TAK-242, 100 nM dose for 0.5 h). Then, cells were collected for ELISA of inflammatory cytokines, followed by quantitative PCR analysis to evaluate inflammatory cytokine levels and gene expression in TAK-242-treated PBLs.

Next, we investigated how AOP works in the presence of LPS. We designed 6 groups: (1) control group (CON), (2) FITC-LPS treatment group (FITC-LPS, 10 μg/mL for 30 min), (3) LPS with FITC-LPS group (LPS + FITC-LPS, simultaneous treatment with LPS and FITC-LPS at 10 μg/mL for 30 min), (4) LPS treatment group (LPS, 10 μg/mL for 30 min), (5) AOP with LPS group (AOP + LPS, treatment with 150 μg/mL AOP and 10 μg/mL LPS for 30 min), and (6) AOP group (AOP, 150 μg/mL for 30 min). Cells were collected for flow cytometry to measure the fluorescence intensity of each treatment group. The excitation and emission wavelengths were 488 nm and 515 nm, respectively. A total of 15,000 cells per sample were analyzed to calculate the average fluorescence intensity. The fluorescence intensities from FITC-LPS, AOP + LPS, and LPS + FITC-LPS treatments were recorded as positive fluorescence. Corresponding background fluorescence was measured for control, AOP-only, and LPS-only treatments. The average fluorescence intensity difference was calculated using the following Equation (1):

Fluorescence intensity of treatment groups = positive fluorescence − corresponding background fluorescence(1)

Lastly, we investigated whether AOP mitigates inflammation through TLR4/NF-κB pathway modulation in PBLs. Using PDTC (an NF-κB p65 inhibitor), cells were assigned to eight treatment groups: (1) control group (CON), (2) AOP group (150 μg/mL AOP, 30 min exposure), (3) AOP with PDTC group (10 μmol/mL PDTC for 30 min, followed by 150 μg/mL AOP), (4) PDTC group (10 μmol/mL PDTC, 30 min exposure), (5) LPS group (10 μg/mL LPS for 6 h), (6) AOP with LPS group (150 μg/mL AOP pretreatment for 24 h, followed by 10 μg/mL LPS for 6 h), (7) LPS with PDTC group (10 μmol/mL PDTC for 30 min, followed by 10 μg/mL LPS for 6 h), and (8) combined PDTC, AOP, and LPS group (10 μmol/mL PDTC and 150 μg/mL AOP pretreatment for 30 min and 6 h, respectively, followed by 10 μg/mL LPS for 6 h). We measured the levels of immune markers [interleukin (IL)-1β, IL-2, IL-4, IL-6, IFN-γ, and immunoglobulins (IgG, IgA, and IgM)] in the culture medium, along with TLR4/NF-κB pathway-related gene expression.

### 3.5. Cell Viability Measurement

Cell proliferation and viability were evaluated using CCK-8 colorimetric assay. The PBLs from each previously described experimental group were seeded into 96-well plates. Following the designated treatment periods, each well received 10 μL of CCK-8 solution, followed by four hours of incubation, after which optical density was measured spectrophotometrically at 490 nm. Cell viability was calculated by subtracting the blank group’s optical density from the treatment group’s optical density, then dividing by the control group’s optical density and multiplying by 100 to express the result as a percentage.

### 3.6. Assay of Immune Indices in Cell Sample

The contents of interleukin (IL) and immunoglobulin (Ig), encompassing IL-1β, IL-6, IL-2, and IL-4, were measured using commercial ELISA kits from Wuhan Colorful Gene Biological Technology (Wuhan, China) according to the manufacturer’s protocol.

### 3.7. RNA Preparation and Fluorescence Quantitative Real-Time PCR

Cellular RNA was isolated directly from cells using Trizol reagent according to protocol. The isolated samples underwent both quantitative and qualitative evaluations by ultraviolet spectrophotometry at A260/A280. To evaluate RNA quality, samples were analyzed by horizontal electrophoresis on 1.5% (*w*/*v*) agarose gels. Removal of genomic DNA contamination was performed using DNase I treatment. The treated RNA was reverse transcribed to cDNA using PrimeScript RT reagent kit with gDNA Eraser (Takara Bio Inc., Otsu, Japan). Quantitative real-time PCR (qPCR) was conducted using TB Green Premix Ex Taq II (Takara Bio Inc.) on an Illumina PCR system. The thermal cycling conditions included initial denaturation at 95 °C for 30 s, followed by 40 cycles of 95 °C for 5 s and 60 °C for 30 s. To verify amplification specificity, melting curve analysis was performed. The genes targeted in this study included toll-like receptor 4 (TLR4), myeloid differentiation primary response 88 (MyD88), nuclear factor kappa-B p65 (NF-κB p65), interleukin-1β (IL-1β), interleukin-6 (IL-6). Primer sequences for these genes were provided by Shanghai Sangon Biotech (Shanghai, China), following the protocols outlined previously [9]. Gene expression was normalized to β-actin and quantified using the 2^−ΔΔCt^ method.

### 3.8. Data Processing and Statistical Analysis

Data distribution normality was tested by PROC UNIVARIATE and homogeneity of variance was assessed by Levene’s test. When data were normally distributed, group comparisons were conducted using one-way ANOVA with honest significant differences determined by Tukey–Kramer post hoc test. All statistical analyses were conducted in SAS (SAS Institute, Cary, NC, USA). Graphs were generated using GraphPad Prism 8.0 (GraphPad Software, Boston, MA, USA) and Adobe Photoshop CS6. The results are expressed as mean ± SEM, with *p* < 0.05 considered statistically significant.

## 4. Conclusions

Collectively, AOP has a molecular weight of 9.00 kDa and contains multiple monosaccharides: glucose (54.08%), arabinose (13.75%), mannose (13.43%), galactose (12.79%), xylose (3.15%), ribose (0.67%), fructose (0.63%), rhamnose (0.56%), and uronic acids including galacturonic and glucuronic acids (0.93%). Additionally, TLR4 is a candidate target for AOP to exert immunomodulatory effects. AOP attenuates LPS-induced immune stress in PBLs by interfering with LPS–TLR binding and suppressing NF-κB pathway activation. Therefore, AOP can be used to improve immune function.

## Data Availability

The raw data supporting the conclusions of this article will be made available by the authors on request.
